# Upadacitinib effectiveness and factors associated with minimal disease activity achievement in patients with psoriatic arthritis: preliminary data of a real-life multicenter study

**DOI:** 10.1186/s13075-023-03182-9

**Published:** 2023-10-11

**Authors:** Michele Maria Luchetti Gentiloni, Valentino Paci, Antonio Carletto, Alen Zabotti, Roberta Ramonda, Maria Sole Chimenti, Lorenzo Dagna, Nicoletta Luciano, Anna Piccinelli, Ivan Giovannini, Giovanni Striani, Nicola Boffini, Gilda Sandri, Niccolò Possemato, Ilenia Pantano, Devis Benfaremo, Carlo Salvarani, Francesco Ciccia, Carlo Selmi, Gianluca Moroncini

**Affiliations:** 1 CLINICA MEDICA, Department of Molecular and Biological Sciences, Marche Polytechnic University, and Department of Internal Medicine, Azienda Ospedaliero Universitaria delle Marche, Ancona, Italy; 2 Internal Medicine Residency Program, Marche Polytechnic University, Ancona, Italy; 3 Department of Medicine, Rheumatology Operative Unit, AOUI Verona, Verona, Italy; 4 Department of Medicine, Rheumatology Institute, University of Udine, Azienda Sanitaria Universitaria Friuli Centrale, Udine, Italy; 5 Department of Medicine-DIMED, Rheumatology Unit, University of Padova, Padua, Italy; 6 Rheumatology, Allergology and Clinical Immunology, University of Rome “Tor Vergata”, Rome, Italy; 7 Unit of Immunology, Allergy and Rare Diseases (UnIRAR), IRCCS San Raffaele Scientific Institute, Milan, Italy; 8 Division of Rheumatology and Clinical Immunology, IRCCS Humanitas Research Hospital, Rozzano, Milan Italy; 9 Department of Maternal, Infantile and Adult Medical and Surgical Sciences, University of Modena and Reggio Emilia, Modena, Italy; 10 Rheumatology Unit, Azienda USL-IRCCS Di Reggio Emilia, Reggio Emilia, Italy; 11 Department of Precision Medicine, Rheumatology Unit, University Della Campania L. Vanvitelli, Naples, Italy; 12 Department of Rheumatology and Clinical Immunology, IRCCS Humanitas Research Hospital, Rozzano, and Department of Biomedical Sciences, Humanitas University, Pieve Emanuele, Milan, Italy

**Keywords:** Psoriatic arthritis, Upadacitinib, Clinical efficacy, Real life, Psoriasis, Peripheral arthritis, Axial inflammation, Minimal disease activity, Bio-refractory, Safety

## Abstract

**Background:**

Upadacitinib (UPA) is a selective JAK inhibitor recently approved for the treatment of psoriatic arthritis (PsA). In this post-approval study, we aimed to evaluate the effectiveness and safety of UPA over 24 weeks and identify clinical predictors of response, in a multicentric cohort of patients affected by PsA.

**Methods:**

One hundred and twenty-six patients with PsA treated with UPA were enrolled in 10 Italian centres. UPA effectiveness outcomes, such as the proportion of patients with MDA status, DAPSA remission, and low disease activity, ASDAS-CRP inactive and low disease activity, and change from baseline in DAPSA and ASDAS-CRP scores, were evaluated every 12 weeks until week 24. The proportion of DAPSA minor, moderate, and major improvement, and ASDAS clinically important improvement (CII) and major improvement (MI) were considered as well. All treatment-related adverse events were collected during the observation period. Clinical predictors of MDA response at week 24 were evaluated through multivariate analysis.

**Results:**

At baseline, 124/126 (98%) and 54/126 (43%) patients showed peripheral and axial involvement, respectively; 110 (87%) patients were intolerant or resistant to biologic DMARDs.

At 24 weeks, MDA status, DAPSA remission, and ASDAS-CRP inactive disease were achieved in 47%, 23%, and 48% of patients, respectively. Minor, moderate, and major DAPSA improvement was observed in 67%, 39%, and 23%, respectively; while 65% and 35% achieved ASDAS-CRP CII and MI, respectively. The mean change from baseline was 15.9 ± 13.5 (*p* < 0.001) for DAPSA and 1.21 ± 0.97 (*p* < 0.001) for ASDAS-CRP. Thirteen patients (10%) discontinued UPA due to a lack of efficacy or non-serious adverse events. No serious adverse events were observed. Male gender (OR 2.54, 95% CI 1.03–6.25 *p* = 0.043), being naïve to biological DMARDs (OR 4.13, 95% CI 1.34–12.71, *p* = 0.013) and elevated baseline CRP (OR 2.49, 95% CI 1.02–6.12, *p* = 0.046) were associated with MDA response at week 24.

**Conclusions:**

This is one of the first real-life studies supporting the effectiveness of UPA and its safety profile in PsA patients. Furthermore, the study identifies predictors of MDA response to UPA treatment at 6 months.

**Supplementary Information:**

The online version contains supplementary material available at 10.1186/s13075-023-03182-9.

## Background

Psoriatic arthritis (PsA) is a chronic systemic inflammatory musculoskeletal disease that affects up to 30% of patients with psoriasis [1–5]. PsA encompasses various phenotypes, including skin and nail psoriasis, peripheral synovitis, enthesitis, dactylitis, and axial involvement. Managing PsA requires considering all these phenotypes based on the 2021 GRAPPA (Group for Research and Assessment of Psoriasis and Psoriatic Arthritis) recommendations [6].

Despite the available therapies, only approximately one-third of patients achieve and maintain minimal disease activity [7]. Thus, there is a significant need for novel therapies effective on as many disease domains as possible, providing personalized treatment for each patient’s phenotypes.

Two oral Janus Kinase (JAK) inhibitors, tofacitinib, and upadacitinib, have been recently approved for use in PsA, with their therapeutic efficacy consistently demonstrated in randomized controlled trials [8–11].

Upadacitinib (UPA) is an oral, reversible JAK inhibitor with higher selectivity for JAK1. The SELECT 1–2 trials have reported its efficacy in various PsA domains, along with significant improvements in pain, fatigue, and quality of life. Safety concerns include opportunistic upper respiratory tract infections and Herpes Zoster Virus (HZV) reactivations [8, 9].

Since real-world data regarding UPA treatment in PsA clinical practice are lacking, we conducted a multicenter observational study to assess its effectiveness and safety over 24 weeks in a cohort of patients with both peripheral and axial PsA.

## Methods

### Study design and objectives

The study coined UPREAL-PsA (UPadacitinib therapy in the REAL-life of patients with PsA) included 10 Italian PsA referral centres and was designed as a prospective cohort study that aims to evaluate clinical responses, adverse events, and laboratory parameters in patients receiving UPA, 15 mg once a day, prescribed according to clinical judgment. The objectives of the study were to (i) describe the characteristics of the patients receiving UPA in daily practice, (ii) evaluate the effectiveness and safety of the treatment over 24 weeks and (iii) identify clinical and demographic predictors of response to UPA.

The data presented here are preliminary, and the study is designed to have at least 1 year of follow-up.

### Subjects

From March 2022, consecutive adult PsA patients with peripheral and/or axial involvement were enrolled in the study if they were prescribed UPA and satisfied the following inclusion criteria: 1) active PsA, defined by the absence of Minimal Disease Activity (MDA) criteria; 2a) failure or intolerance towards at least one conventional synthetic (cs-) disease-modifying anti-rheumatic drug (DMARD) for bio-naïve patients, or 2b) failure of at least one biological (b-) or targeted synthetic (ts-) DMARD (bio-failure). Patients were excluded if: women of childbearing potential not willing to use adequate contraceptive methods or pregnant; recent history of malignant neoplasia; active infections, including chronic hepatitis B or latent tuberculosis; severe kidney or liver failure; uncontrolled cardiovascular disease and/or medical history of major cardiovascular adverse events.

PsA diagnosis was based on the investigators’ judgment and by fulfilment of the ClASsification criteria for Psoriatic Arthritis (CASPAR) criteria [12]. Axial involvement of PsA was diagnosed based on investigators’ judgment and by fulfilment of the Assessment of Spondyloarthritis International Society (ASAS) classification criteria for axial spondyloarthritis (SpA) [13]; patients with exclusive spine involvement, defined by active lesions on MRI fulfilling OMERACT criteria [14, 15], were considered to be affected by axial PsA as well.

The study protocol was approved by the CERM (Comitato Etico Regionale delle Marche, Azienda Ospedaliero Universitaria delle Marche, Ancona, Italy) and fell under good clinical practice.

### Clinical and laboratory features

For all patients, we gathered data on gender, age, body mass index (BMI), time of diagnosis of PsA and disease duration, previous conventional and biologic / targeted synthetic DMARD. A full list of comorbidities was collected at UPA initiation, including: a. cardiovascular diseases, such as hypertension, ischemic cardiomyopathy, heart failure, cardiac arrhythmias (any), valvular disease (any), stroke, venous thromboembolism, pericardial disease, and other cardiomyopathies; b. metabolic diseases, such as obesity, diabetes type I and II, dyslipidaemia, and osteoporosis; c. depression and/or anxiety disorders; d. neoplastic diseases, including solid cancer, haematological cancer, and non-melanoma skin cancer; e. other comorbidities not listed above (see Additional Table 2).

Clinical data were obtained at baseline, week 12, and week 24 in all patients, including tender joint count 68 and swollen joint count 66 (TJC68/SJC66), Leeds Enthesitis Index (LEI), Psoriasis Area Severity Index (PASI), Disease Activity Index for Psoriatic Arthritis (DAPSA), Health Assessment Questionnaire (HAQ), Visual Analogue Scale for pain (VAS pain), Patient and Physician Global Assessment (PtGA and PGA).

Patients with confirmed axial involvement of PsA were also clinically assessed using the Ankylosing Spondylitis Disease Activity Score With C-Reactive Protein (ASDAS-CRP).

Laboratory data, including complete blood count, liver and kidney function test, erythrocyte sedimentation rate (ESR), and C-reactive protein (CRP), were collected at the same time points. Any adverse events, either assessed by the physician or reported by the patient, were recorded and classified using the Common Terminology Criteria for Adverse Events (CTCAE) version 5.0.

### Outcomes

The primary effectiveness outcome was the proportion of patients who achieved MDA [16] and Very Low Disease Activity (VLDA) [17] status at week 24. The primary safety outcome was the proportion of serious adverse events (SAE) during the observation period.

The secondary effectiveness outcome was the proportion of patients who achieved a clinically relevant response in DAPSA and ASDAS-CRP scores. Regarding DAPSA: minor, moderate, and major responses were defined as a 50%, 75%, and 85% change from baseline, respectively [18]. Regarding ASDAS-CRP: clinically important improvement (CII) and major improvement (MI) was defined as a decrease of at least 1.1 and 2.0 points from baseline, respectively [19]. Furthermore, the proportions of patients achieving DAPSA remission and low disease activity, as well as ASDAS-CRP inactive disease or low disease activity, were evaluated as well [18, 19].

### Data analysis

Study data were collected and managed using the REDCap electronic data capture tool hosted at “Azienda Ospedaliero Universitaria delle Marche” (www.project-redcap.org). REDCap (Research Electronic Data Capture) is a web-based software platform designed to support data capture for research studies [20, 21].

For descriptive analyses, categorical variables were reported as absolute number and frequency, continuous variables as mean and standard deviation or median and interquartile range according to their distribution. The normality of distribution was assessed by Kolmogorov-Smirnov test.

The effectiveness of UPA was assessed by the proportion of patients achieving MDA at week 12 and week 24, and by the proportion of patients achieving a clinically relevant response in DAPSA and ASDAS composites scores, as defined in the *“*Outcomes*”* section. The differences between DAPSA and ASDAS scores at baseline and weeks 12 and 24 were also evaluated, using the Student t-test for paired samples.

The safety of UPA was assessed by the proportion of patients experiencing adverse events, particularly SAE, during the observation period.

The correlations between variables were analyzed using the Pearson’s correlation coefficient. A multivariate regression analysis with stepwise selection was performed to identify predictors of MDA at week 24. The results of the regression analysis were expressed as odds ratio (OR) and 95% confidence interval.

All statistical analyses were performed using the STATA software, version 14.0. The GraphPad Prism software, version 9.5.1, was used to generate the Figures. A *p*-value ​​less than 0.05 was considered significant.

## Results

### Baseline characteristics

A total of 126 patients were enrolled, including 86 (68%) women. Mean age was 57 ± 11 years, mean BMI 26.7 ± 5.1 kg/m^2^, and median duration (InterQuartile Range, IQR) of the disease was 92 (45–177) months.

Most patients (124/126, 98%) had peripheral joint involvement, with 39 (31%) and 85 (68%) oligoarticular and polyarticular patterns, respectively. Axial involvement was found in 54 (43%) patients: however, only two patients showed isolated axial involvement whereas the others were also affected by peripheral joint disease.

Baseline characteristics of the patients are shown in Table 1, and in Additional Fig. 1—Additional Tables 1a-b, and 2.

**Table 1:** Characteristics of the UPREAL-PsA study patients at baseline

PsA Patients	Total number (%)
**Gender, female**	86 (68.3)
**Gender, male**	40 (31.7)
**Age, years ± SD**	56.5 ± 11.4
**BMI, value ± SD**	26.7 ± 5.1
**Cardiovascular Diseases**^**a**^	40 (31.8)
**Hypertension**	27 (21.4)
**Metabolic Diseases**^**b**^	32 (25.4)
**Obesity**	23 (18.3)
**Diabetes type II**	12 (9.5)
**Depression/Anxiety Disorder**	18 (14.3)
**Neoplastic Diseases**^**c**^	7 (5.6)
**Other Comorbidities**^**d**^	98 (77.8)
**Peripheral PsA**	124 (98.4)
**Axial PsA**	54 (42.9)
**Enthesitis**	69 (54.8)
**Dactylitis**	30 (23.8)
**Skin**	78 (61.9)
**Nails**	44 (34.9)
**Uveitis (ever)**	6 (4.8)
**IBD (ever)**	6 (4.8)
**DAPSA at baseline,** mean ± SD	27.7 ± 10.0
**ASDAS-CRP at baseline**^e^**,** mean ± SD	2.65 ± 0.69
**Disease Duration,** months median (IQR)	92 (45–177)

Clinical features of patients’ cohort in UPREAL-PsA (Upadacitinib therapy in the real-life in patients with psoriatic arthritis)

*PsA* Psoriatic Arthritis, *PsO* Psoriasis, *BMI* Body Mass Index, *Oligo* Oligoarticular involvement, *Poly* Polyarticular Involvement, *IBD* Inflammatory Bowel Disease, *DAPSA* Disease Activity in Psoriatic Arthritis, *ASDAS-CRP* Ankylosing Spondylitis Disease Activity Score with the C-reactive protein, *SD* Standard Deviation, *IQR* Interquartile Range

^a^Ischemic Cardiomyopathy, Heart Failure, Cardiac Arrhythmias (any), valvular disease (any), Stroke, Venous Thromboembolism, Pericardial Disease, other Cardiomyopathies

^b^Diabetes type I, Dyslipidaemia, Osteoporosis

^c^Solid Cancer. Haematological Cancer, Non-Melanoma Skin Cancer

^d^See Additional Table 2

^e^Only in patients with axial PsA (*n* = 54)

Most patients (109/126, 87%) were bio-failure with 19 (17%) having failed one line, 27 (25%) two lines, and 63 (58%) three or more lines of biologic or targeted synthetic DMARDs. The most common previous biological DMARD failed was adalimumab (70%), followed by etanercept (53%) and secukinumab (53%), as illustrated in Additional Table 3.

Among the whole cohort, 34 patients (27%) were taking a csDMARD at baseline (25 methotrexate, 7 sulfasalazine, 2 leflunomide) and continued the same dose throughout the observation period.

At baseline, all patients showed a high disease activity with mean DAPSA of 27.7 ± 10.0. Patients with axial PsA showed mean ASDAS-CRP of 2.65 ± 0.69. Skin psoriasis was active in 52/126 (41%) patients at baseline, generally with low PASI scores (mean 1.34 ± 4.17) (Tables 1 and 2 – Additional Table 1a-b).

**Table 2:** Clinical parameters, clinimetric test, and clinical response to the therapy in UPREAL-PsA study patients

	**Baseline**	**w12**	**p**^**a**^	**w24**	**p**^**b**^
**Nr. of patients (%)**	126 (100.0)	97 (77.0)	/	66 (52.4)	/
**Disease-Clinimetric Indexes**					
**Tender Joint Count, mean ± SD**	9.36 ± 6.76	4.05 ± 4.70	** < 0.001**	3.59 ± 6.35	** < 0.001**
**Swollen Joint Count, mean ± SD**	3.02 ± 3.06	1.09 ± 2.53	** < 0.001**	0.86 ± 2.19	** < 0.001**
**Leeds Enthesitis Index, mean ± SD**	0.91 ± 1.41	0.33 ± 0.78	** < 0.001**	0.32 ± 0.77	**0.0033**
**PASI, mean ± SD**	1.34 ± 4.17	0.42 ± 1.06	**0.0094**	0.69 ± 3.15	**0.0373**
**PtGA (scale 0–100) mean ± SD**	71.99 ± 16.6	43.42 ± 20.9	** < 0.001**	36.06 ± 21.6	** < 0.001**
**PGA (scale 0–100) mean ± SD**	63.11 ± 16.2	31.92 ± 21.1	** < 0.001**	28.56 ± 23.8	** < 0.001**
**VAS (scale 0–100) mean ± SD**	71.21 ± 18.0	42.50 ± 24.7	** < 0.001**	33.11 ± 23.0	** < 0.001**
**HAQ, mean ± SD**	1.24 ± 0.90	0.66 ± 0.78	0.0709	0.44 ± 0.56	**0.0075**
**C-Reactive Protein, mg/dl**	0.99 ± 1.80	0.40 ± 0.75	** < 0.001**	0.35 ± 0.83	** < 0.001**
**Composite Scores, global**					
**MDA** nr. (%)	0 (0.0)	29 (30.9)	/	31 (47.0)	/
**VLDA** nr. (%)	0 (0.0)	9 (9.6)	/	9 (13.6)	/
**DAPSA, mean ± SD**	27.65 ± 10.0	14.05 ± 9.48	** < 0.001**	11.77 ± 10.7	** < 0.001**
**DAPSA minor improvement**, nr. (%)	/	47 (48.5)	/	44 (66.7)	/
**DAPSA moderate improvement**, nr. (%)	/	23 (23.7)	/	26 (39.4)	/
**DAPSA major improvement**, nr. (%)	/	12 (12.4)	/	15 (22.7)	/
**DAPSA LDA**, nr. (%)	0 (0.0)	41 (42.3)	/	35 (53.0)	/
**DAPSA remission status**, nr. (%)	0 (0.0)	14 (14.4)	/	15 (22.7)	/
**Composite Scores, axial**					
**Nr. of patients with axial involvement (%)**	54 (100)	38 (70.4)	/	23 (42.6)	/
**ASDAS-CRP, mean ± SD**	2.65 ± 0.69	1.71 ± 0.79	** < 0.001**	1.43 ± 0.78	**0.0019**
**ASDAS-CRP CII**, nr. (%)	/	18 (47.4)	/	15 (65.2)	/
**ASDAS-CRP MI**, nr. (%)	/	4 (10.5)	/	8 (34.8)	/
**ASDAS-CRP LDA status**, nr. (%)	0 (0.0)	12 (31.6)	/	9 (39.1)	/
**ASDAS-CRP inactive disease status**, nr. (%)	0 (0.0)	12 (31.6)	/	11 (47.8)	/

Variation of clinical parameters and clinimetric tests in response to the therapy in UPREAL-PsA (Upadacitinib therapy in the real-life in patients with psoriatic arthritis) study at week 12 (w12) and 24 (w24)

*PASI* Psoriasis Area Severity Index, *PtGA* Patient Global Assessment, *PGA* Physician Global Assessment, *VAS pain* Visual Analogue Scale for pain, *HAQ* Health Assessment Questionnaire, *DAPSA* Disease Activity in Psoriatic Arthritis, *ASDAS-CRP* Ankylosing Spondylitis Disease Activity Score/C-reactive protein, *MDA or VLDA* Minimal or Very Low Disease Activity, respectively, *LDA* low disease activity, *CII* clinically important improvement, *MI* major improvement. Statistical analysis conducted by “Stata” software (Paired Student’s *t* test test). *P*-value significant (in bold) if < 0.05; p^a^ between baseline and w12 and p^b^ between w12 vs w24

Comparison of patient subgroups showed that cardiovascular disease was significantly associated with axial involvement while metabolic disease was associated with polyarticular involvement; patients with axial involvement had longer disease duration than peripheral involvement (Additional Table 1a-b).

Furthermore, patients with polyarticular involvement, metabolic comorbidities, higher body-mass index, and elevated CRP showed significantly higher disease activity scores at baseline, as assessed by DAPSA (Additional Table 1a-b).

### Follow up

The study is ongoing and, at the time of the analysis, 97/126 (77%) and 66/126 (52%) patients have reached the week 12 and week 24 assessments, respectively. Currently, no patients have been lost to follow-up.

During the study, 7 (6%) patients discontinued UPA due to secondary ineffectiveness: 3 at week 12 and 4 at week 24. Additionally, 6 (5%) patients discontinued UPA due to adverse events (see below).

### Effectiveness of UPA at 12 and 24 weeks

**Fig. 1:**
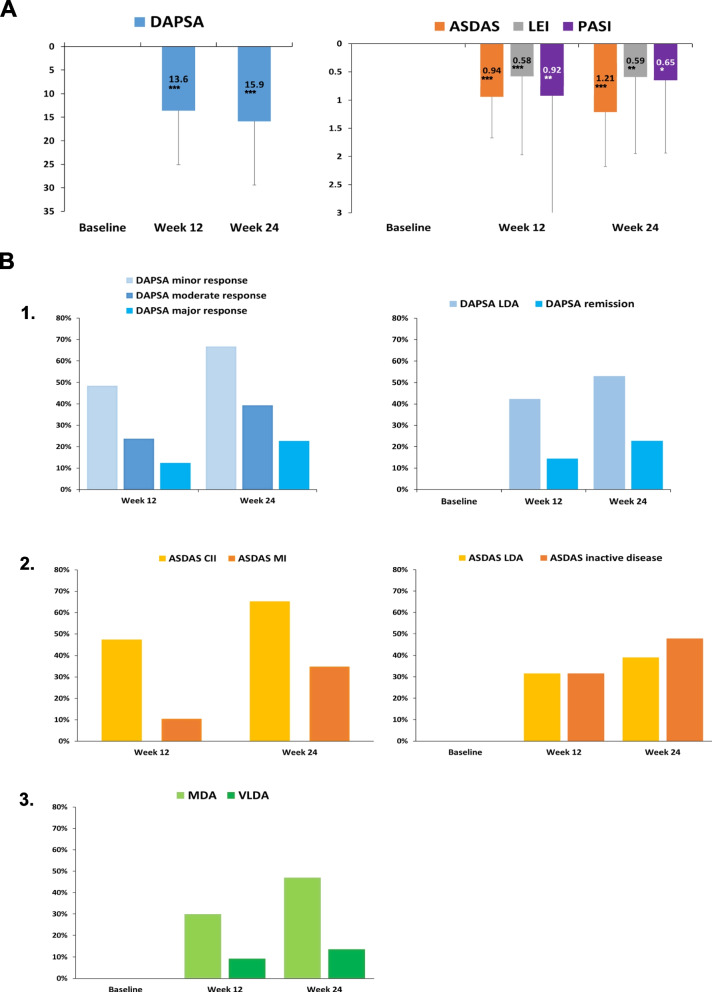
Clinical responses to the therapy in the UPREAL-PsA study. **A** from left to right: change from baseline in the DAPSA (Disease Activity in Psoriatic Arthritis), ASDAS-CRP (Ankylosis Spondylitis Disease Activity Score with C-reactive protein), LEI (Leeds Enthesitis Index), and PASI (Psoriasis Area Severity Index) numerical values in response to the therapy with upadacitinib. **B** from left to right: 1) Proportion (%) of the patients achieving DAPSA minor, moderate, and major response and DAPSA LDA (Low Disease Activity) and remission status; 2) Proportion of patients achieving ASDAS CII (Clinical Important Improvement) and MI (Major Improvement), and ASDAS LDA and inactive disease status; 3) Proportion of patients achieving MDA or VLDA (Minimal or Very Low Disease Activity, respectively) in response to the therapy with upadacitinib. The results are shown at baseline, week 12 (w12), and week 24 (w24). Statistical analysis was conducted using the “Stata” software. Statistical significance: *p* < 0.05^*^; *p* < 0.01^**^; *p* < 0.001^***^

The MDA and VLDA status were achieved respectively in 29/97 (31%) and 9/97 (10%) patients at week 12, and respectively in 31/66 (47%) and 9/66 (14%) patients at week 24 (Table 2 and Fig. 1B-3).

At both timepoints, change from baseline was significant for DAPSA (13.6 ± 11.5 and 15.9 ± 13.5, respectively, *p* < 0.001 for both comparisons) and for ASDAS-CRP (0.94 ± 0.73 and 1.22 ± 0.97, respectively, *p* < 0.001 for both comparisons), as illustrated in Table 2, and Fig. 1A.

DAPSA minor, moderate and major response were achieved respectively in 44/66 (67%), 26/66 (39%), and 15/66 (23%) patients at week 24. DAPSA low disease activity and remission status were achieved respectively in 35/66 (53%) and 15/66 (23%) at week 24 (Table 2, Fig. 1B-1).

**Fig. 2:**
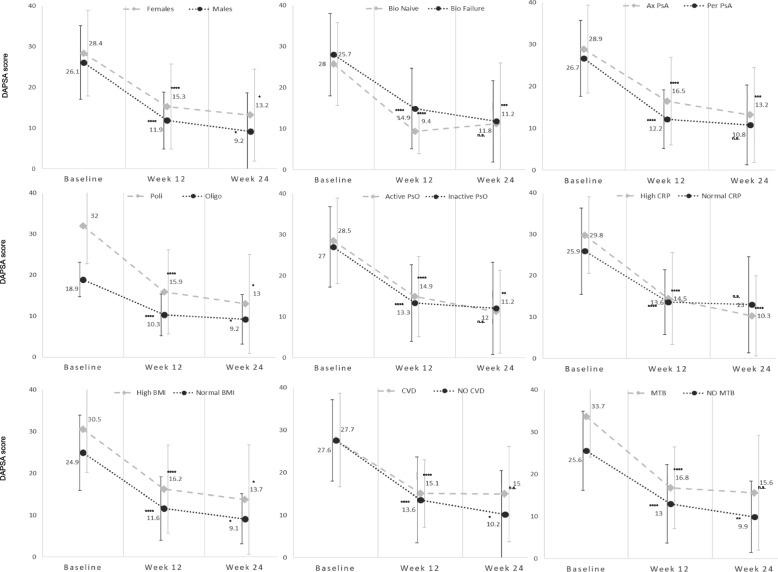
DAPSA subgroups responses to Upadacitinib in the UPREAL-PsA study. DAPSA (Disease Activity in Psoriatic Arthritis) in the UPREAL-PsA (Upadacitinib therapy in the real-life in patients with psoriatic arthritis) study at week 12 (w12) and 24 (w24) in different patients’ subgroups: Bio-Naïve and Bio-Failure: naïve to or treated with biological drugs; AxPsA and PerPsA: axial (prevalent) or peripheral inflammation; Poli and Oligo: polyarticular or oligoarticular involvement; PsO: Psoriasis; Norm-CRP vs High-CRP: C-Reactive Protein normal or upper the normal limit (0.05 mg/dl); High- vs normal-BMI: body mass index > 30 o < 30, respectively; CVD: cardiovascular diseases; MTB: metabolic diseases. Statistical analisys was performed between DAPSA at week 12 and baseline and between DAPSA at week 24 and week 12. Statistical analysis was conducted by “Stata” software (Paired Student’s *t* test). Statistical significance: n.s.: not significant; *p*: < 0.05^*^, < 0.01^**^, < 0.001^***^, < 0.0001^****^

ASDAS clinically important improvement and major improvement were achieved respectively in 15/23 (65%) and 8/23 (35%) patients with axial involvement at week 24. ASDAS low disease activity and inactive disease status were achieved respectively in 9/23 (39%) and 11/23 (48%) at week 24 (Table 2, Fig. 1B-2).

In parallel with the composite clinical scores, all clinical indices (i.e., TJC, SJC, LEI, PASI, PtGA, PGA, VAS pain), functional status (assessed by HAQ) and CRP showed a significant amelioration at week 24 (Table 2, and Fig. 1A).

### Subgroup analysis

When analysing patients’ subgroups in terms of DAPSA response, the normal CRP group experienced a rapid clinical improvement starting from week 12, and this improvement was maintained at week 24 (DAPSA 13.6 ± 7.8 and 13.0 ± 11.6, respectively: *p* = n.s.;). The high CRP group, which showed significantly higher DAPSA at baseline, also experienced a clinical improvement at week 12. However, unlike the normal CRP group, they continued to show a further significant improvement from week 12 to week 24 (DAPSA 14.5 ± 11.1 and 10.3 ± 9.7, respectively: *p* < 0.0001;), reaching numerically lower scores to the normal CRP group (Fig. 2, Additional Table 4).

Similar results also emerged for the oligoarticolar and bio-naïve groups, compared to the polyarticular and bio-failure groups, with the latter showing significant further clinical improvement from weeks 12 to 24.

When considering patients with cardiovascular or metabolic comorbidities, these groups showed clinical improvement at week 12, which was maintained at week 24. On the other hand, patients without comorbidities continued to clinically improve from week 12 to week 24 (Fig. 2, Additional Table 4).

Regarding gender subgroups, both female and male patients showed significant clinical improvement at week 12 and continue to experience further significant improvement from week 12 to week 24 (Fig. 2, Additional Tables 4 and 5). Furthermore, all the subgroups of axial PsA patients showed a significant reduction from baseline in the ASDAS score, except for the Bio-Naïve group, where the differences were not statistically significant due to the small sample size (Additional Table 5).

Finally, we observed that patients who have failed two or more lines of previous biologic DMARDs have similar differences from baseline in DAPSA score at both timepoints (13.7 ± 12.7 and 16.3 ± 14.1, respectively, *p* < 0.001 for both comparisons), compared with the whole cohort. However, in this subgroup of patients, the MDA and VLDA response were achieved in a numerically lesser proportion of patients compared to the whole cohort (22.8% vs 30.9% and 5.7% vs 9.6% at week 12; 34.1% vs 47.0% and 11.4% vs 13.6% at week 24, respectively; p not significant for all the comparisons); statistical analysis was not significant due to the small size of the sample.

To identify predictors of MDA response, we conducted a multivariate logistic regression analysis, which showed that male gender (OR 2.54, 95% CI 1.03–6.25 *p* = 0.043), bio-naïve status (OR 4.13, 95% CI 1.34–12.71, *p* = 0.013) and high baseline CRP (OR 2.49, 95% CI 1.02–6.12, *p* = 0.046) were associated with achieving MDA at week 24 (Table 3).

**Table 3:** Multivariate logistic regression analysis

	**Odds Ratio**	**95% Confidence Interval**	***p* value**
**Gender**			
Male vs Female	2.537	1.030 - 6.247	***0.043***
**CRP**			
High vs Normal	2.493	1.016 - 6.115	***0.046***
**Bio-DMARD**			
Naive vs Failure	4.128	1.340 –12.708	***0.013***
**Psoriasis**			
Active vs Inactive	0.462	0.188 –1.133	0.092

Results of the multivariate logistic regression analysis showing predictors of achieving MDA at week 24. CRP, C Reactive Protein normal or upper (High) the normal limit (0.5 mg/dl); Bio-DMARD, biological Disease Modifying Anti-Rheumatic Drug naïve or refractory (Failure) to at least one biologic DMARDs. *p*-value was considered significant if < 0.05 (shown in bold)

### Safety of UPA at 12 and 24 weeks

The overall period of observation accounted for 52.5 patient years. During this period, no life-threatening serious adverse event was observed. In total, there were 21 adverse events observed in 17 patients (14%), with the most common being gastrointestinal (2 diarrhea, 1 nausea, 1 dyspepsia) and infections (2 HSV reactivations, 2 urinary tract infections, 2 COVID-19, 1 sinusitis).

Minor laboratory abnormalities included liver enzymes elevation in 2 patients, thrombocytopenia in 2 patients, neutropenia in 1 patient, and creatine kinase elevation in 2 patients.

UPA therapy was stopped for adverse events in 6 (5%) patients, 2 for moderate thrombocytopenia, 2 for creatine phosphokinase elevation, 1 for severe hypertension, 1 for diarrhea, nausea, and dyspepsia with mild hypotension (Additional Table 6); in none of these cases, patients required hospital admission.

## Discussion

PsA is a chronic immune-mediated musculoskeletal disease that is considered a systemic disease for its heterogenic clinical presentation and for being tightly correlated to several comorbid diseases like cardiovascular, metabolic, infective, psychological, and neoplastic ones [22–25]. As such, growing evidence shows that disease activity and therapeutic response vary considerably among patients with peripheral and/or axial involvement and with the number and/or type of extra-articular domains involved [25–30].

Two JAK inhibitors have been recently licensed for use in PsA, following the results of randomized controlled trials (RCTs) that demonstrated their efficacy in several disease domains [8–11, 31–35]. However, the discrepancy between RCTs and real-world evidence can be significant, in some cases, given the use of new medications in patients that would not be eligible for phase III studies.

Herein, we reported preliminary results from an ongoing multicenter prospective cohort study that aims to illustrate the real-life efficacy and safety of upadacitinib (UPA) therapy in patients with PsA (UPREAL-PsA study) and determine factors influencing clinical outcomes at 6 months.

Our data show that UPA is effective already at 12 weeks and maintains activity at 24 weeks, both in the composite and in the single domains scores (peripheral, enthesis, and skin ones), in agreement with the results of RTCs.

Notably, UPA demonstrated consistent efficacy in patients with axial involvement up to the 24-week follow-up. In this regard, it should be underlined that there is growing interest in the assessment of axial symptoms in PsA patients, and an interesting debate is ongoing on whether axial-PsA should be considered only axial-SpA with psoriasis or a separate clinical entity [36–38]. Therefore, we believe that an important added value of our cohort is that patients were defined as having axial PsA when both the CASPAR criteria and the ASAS classification criteria for axial SpA were met [12, 13], and this allowed us to evaluate the benefits of UPA in this unique PsA phenotype.

In comparison to the UPA 15 mg arm of the SELECT 1 RCT [8], our study shows a higher proportion of female patients (68.3% vs. 55.5%), a slightly higher mean age (56.5 ± 11.4 vs. 51.6 ± 12.2), and a longer duration of disease (10.0 ± 8.3 vs. 6.2 ± 7.4 years). Furthermore, our study included a significant proportion of patients who were bio-experienced, which were excluded from the SELECT 1 trial. Lastly, the proportion of patients achieving MDA is comparable between the two studies.

Very recently, another real-life study on the effectiveness of UPA in PsA patients from Germany and Canada was published [39]. The results of this study are similar to those of our own conducted in Italy, and the data from these different populations mutually support each other.

Our study also showed results comparable to a real-life study on the effectiveness of Tofacitinib in PsA [40]. The reduction in DAPSA score at 24 weeks aligns with our study, showing similar outcomes. A notable difference is that none of our patients received steroid therapy during the study.

Of note, patients having a worse global health state at baseline (such as elevated BMI with metabolic comorbidities; debilitating long-lasting PsA such as those with more involved skin and/or higher number of inflamed joints) were also more likely to present higher PsA disease activity at baseline. Interestingly, UPA was effective in reducing clinical activity, as assessed by DAPSA and ASDAS-CRP, in all the patient subgroups, but the results of the multivariate analysis suggested that bio-naïve patients and those with higher CRP at baseline were more likely to achieve the dichotomous MDA response at week 24.

Finally, the regression analysis also showed that males were more likely to achieve MDA at week 24 compared to females. These data corroborate similar recent findings supporting the need for a gender-specific evaluation and therapeutic strategy adjustment in PsA patients [41–44]. In this regard, it should be emphasized that subjective parameters (i.e., PtGA, VAS pain, TJC, HAQ) tend to be worse than the objective ones (i.e. PhGA, SJC, LEI, PASI, CRP), suggesting that the inadequate response of some subgroups of patients could be related, at least in part, to a negative perception of their global health status and central pain processing mechanisms, i.e. nociplasticity [45].

In this study, the safety profile of UPA was reassuring, based on the limited number of adverse events of any type, the absence of serious adverse events (including thromboembolic or major cardiovascular events), and the low number of patients lost at the 24-week follow-up.

In our opinion, this study has several strengths. First, this is one of the first real-life studies conducted in a large cohort of heterogeneous PsA patients and provides a comprehensive assessment of PsA patients initiating UPA in real-life. Second, we identified clinical and demographic parameters associated with a better response to UPA therapy in a real-life cohort of PsA patients.

We are also aware of the limitations of our study. First, the study is observational, and it has been conducted only in tertiary referral centers in a single country (Italy), thus it may not fully represent the entire spectrum of the disease.Second, due to the ongoing nature of the study and the preliminary nature of the data, the short-term observation period may lead to an underestimation of the incidence of adverse events or patient dropouts. Additionally, it does not allow for the assessment of survival. Third, the proportion of bio-naïve patients with early-stage disease is relatively small: most patients have previously failed at least one b-DMARD, and half of them have failed more than three biological lines, which can affect drug responsiveness. In this context, it is important to note that regulatory restrictions in our country limit the use of JAK inhibitors in bio-naïve patients, and these limitations may introduce a slight bias in the composition of our real-life study cohort. Fourth, we acknowledge that certain confounding factors, such as the use of non-steroidal anti-inflammatory drugs or the dose of methotrexate, were not specifically recorded and therefore not accounted for in our analysis.

## Conclusions

The 24-week results from the UPREAL-PsA study confirm the remarkable efficacy and good safety profile of upadacitinib therapy in real-life PsA, with the higher efficacy of UPA demonstrated in males, bio-naïve patients, and those with elevated baseline CRP.

### Supplementary Information

**Additional file 1: Additional Table 1a.** Characteristics of the UPREAL-PsA study patients subgroups at baseline. **Additional Table 1b.** Characteristics of the UPREAL-PsA study patients subgroups at baseline.** Additional Table 2.** List of the comorbidities considered as Other in patients of the UPREAL-PsA study. **Additional Table 3.** List of the previous bDMARDs used in bio-failure patients. **Additional Table 4.** DAPSA responses in patients’ subgroups of the UPREAL-PsA study.** Additional Table 5.** ASDAS-CRP responses in patients’ subgroups of the UPREAL-PsA study.** Additional Table 6.** List of all the adverse events observed in patients of the UPREAL-PsA study.

**Additional file 2: Additional Figure 1.** Characteristics of the UPREAL-PsA patients at baseline. Graphical representation of the most representative clinical features of the patient’s cohort of the UPREAL-PsA (Upadacitinib therapy in the REAL life of patients with psoriatic arthritis) study. Bio-naïve: patients treated>3 months with conventional synthetic Disease-Modifying Anti-Rheumatic Drugs (csDMARDs); Bio-Failure: patients refractory to at least one biologic DMARDs; PsO: Psoriasis; Norm- or High-CRP: C- Reactive Protein normal or upper the normal limit (0.05 mg/dl); High-BMI and normal BMI: patients with body mass index>30 o <30, respectively; CVD: cardiovascular comorbidities and MTB: Metabolic Diseases: see the full list in Table 1.

## Data Availability

The datasets used and/or analysed during the current study are available from the corresponding author on reasonable request.

## Abbreviations

PsA: Psoriatic arthritis
UPA: Upadacitinib
JAK: Janus Kinase
GRAPPA: Group for Research and Assessment of Psoriasis and Psoriatic Arthritis
UPREAL-PsA: (UPadacitinib therapy in the REAL-life of patients with PsA) study
CASPAR: ClASsification for Psoriatic ARthritis criteria
ASAS: Assessment of Spondyloarthritis International Society
DMARDs: Disease-modifying anti-rheumatic drugs
TJC: Tender joint count
SJC: Swollen joint count
LEI: Leeds Enthesitis Index
PASI: Psoriasis Area Severity Index
DAPSA: Disease Activity Index for Psoriatic Arthritis
ASDAS-CRP: Ankylosing Spondylitis Disease Activity Score With C-Reactive Protein
HAQ: Health Assessment Questionnaire
VAS-pain: Visual Analogue Scale for pain
PtGA: Patient Global Assessment
PhGA: Physician Global Assessment
MDA: Minimal Disease Activity
VLDA: Very Low Disease Activity
ESR: Erythrocyte sedimentation rate
CRP: C-reactive protein
