# Supplementary material for: Upadacitinib effectiveness and factors associated with minimal disease activity achievement in patients with psoriatic arthritis: preliminary data of a real-life multicenter study
Source: Arthritis Res Ther. 2023 Oct 11;25:196. doi: 10.1186/s13075-023-03182-9 (PMC10565976; doi:10.1186/s13075-023-03182-9)
Supplement: Supplementary file 1 — Additional file 1: Additional Table 1a. Characteristics of the UPREAL-PsA study patients subgroups at baseline. Additional Table 1b. Characteristics of the UPREAL-PsA study patients subgroups at baseline. Additional Table 2. List of the comorbidities considered as Other in patients of the UPREAL-PsA study. Additional Table 3. List of the previous bDMARDs used in bio-failure patients. Additional Table 4. DAPSA responses in patients’ subgroups of the UPREAL-PsA study. Additional Table 5. ASDAS-CRP responses in patients’ subgroups of the UPREAL-PsA study. Additional Table 6. List of all the adverse events observed in patients of the UPREAL-PsA study. [file 13075_2023_3182_MOESM1_ESM.docx]

**Addictional Table 1a.** **Characteristics of the UPREAL-PsA study patients subgroups at baseline**.

| **PsA Patients**  **Total number (%)** | | **Females**  **86 (68.3)** | **Males**  **40 (31.7)** | **p** | **Bio-Naive 17 (13.5)** | **Bio-Failure**  **109 (86.5)** | **p** | **Ax (±Per)**  **54 (42.9)** | **Per (only)**  **72 (57.1)** | **p** | **Oligo**  **39 (31.0)** | **Poly**  **85 (67.5)** | **p** | **Active PsO**  **52 (41.3)** | **Inactive PsO**  **74 (58.7)** | **p** |
| --- | --- | --- | --- | --- | --- | --- | --- | --- | --- | --- | --- | --- | --- | --- | --- | --- |
| **Gender, female** | 86 (68.3) | 86 (100) | 0 | <0.01 | 13 (76.5) | 73 (67.0) | 0.434 | 41 (75.9) | 45 (62.5) | 0.109 | 24 (61.5) | 61 (71.8) | 0.255 | 33 (63.5) | 53 (71.6) | 0.333 |
| **Gender, male** | 40 (31.7) | 0 (0) | 40 (100) | <0.01 | 4 (23.5) | 36 (33.0) | 0.434 | 13 (24.1) | 27 (37.5) | 0.109 | 15 (38.5) | 24 (28.2) | 0.255 | 19 (36.5) | 21 (28.4) | 0.333 |
| **Age, years ± SD** | 56.5±11.4 | 56.4±11.8 | 56.5±10.7 | 0.424 | 57.5±13.1 | 56.3±11.2 | 0.315 | 56.2±10.5 | 56.6±12.1 | 0.482 | 56.4±10.7 | 56.4±11.5 | 0.428 | 56.1±11.4 | 56.7±11.5 | 0.457 |
| **BMI, value ± SD** | 26.7±5.1 | 26.2±4.9 | 27.8±5.3 | 0.057 | 24.4±3.6 | 27.1±5.2 | **0.027** | 27.1±5.1 | 26.4±5.0 | 0.220 | 25.5±5.4 | 27.3±4.9 | **0.038** | 27.0±5.2 | 26.5±5.0 | 0.309 |
| **Cardiovascular Diseases^a^** | 40 (31.8) | 27 (31.4) | 13 (32.5) | 0.451 | 4 (23.5) | 36 (33.0) | 0.219 | 23 (42.6) | 17 (23.6) | **0.012** | 13 (33.3) | 25 (29.4) | 0.331 | 16 (30.8) | 24 (32.4) | 0.422 |
| **Hypertension** | 27 (21.4) | 20 (23.3) | 7 (17.5) | 0.464 | 3 (17.7) | 24 (22.0) | 0.683 | 15 (27.8) | 12 (16.7) | 0.133 | 10 (25.6) | 16 (18.8) | 0.387 | 8 (15.4) | 19 (25.7) | 0.166 |
| **Metabolic Diseases^b^** | 32 (25.4) | 23 (26.7) | 9 (22.5) | 0.307 | 2 (11.8) | 30 (27.5) | 0.083 | 16 (29.6) | 16 (22.2) | 0.174 | 3 (7.7) | 27 (31.7) | **<0.01** | 12 (23.1) | 20 (27.0) | 0.309 |
| **Obesity** | 23 (18.3) | 14 (16.3) | 9 (22.5) | 0.400 | 1 (5.9) | 22 (20.2) | 0.154 | 10 (18.5) | 13 (18.1) | 0.947 | 5 (12.8) | 18 (21.2) | 0.266 | 10 (19.2) | 13 (17.6) | 0.812 |
| **Diabetes type II** | 12 (9.5) | 8 (9.3) | 4 (10.0) | 0.901 | 0 (0.0) | 12 (11.0) | 0.150 | 6 (11.1) | 6 (8.3) | 0.599 | 2 (5.1) | 10 (11.8) | 0.246 | 6 (11.5) | 6 (8.1) | 0.518 |
| **Depression/Anxiety Disorder** | 18 (14.3) | 13 (15.1) | 5 (12.5) | 0.964 | 2 (11.8) | 16 (14.7) | 0.901 | 11 (20.4) | 7 (9.7) | 0.089 | 6 (15.4) | 12 (14.1) | 0.447 | 5 (9.6) | 13 (17.6) | 0.384 |
| **Neoplastic Diseases^c^** | 7 (5.6) | 4 (4.7) | 3 (7.5) | 0.260 | 0 (0.0) | 7 (6.4) | 0.143 | 4 (7.4) | 3 (4.2) | 0.218 | 4 (10.3) | 3 (3.5) | 0.067 | 4 (7.7) | 3 (4.1) | 0.192 |
| **Other Comorbidities^d^** | 98 (77.8) | 69 (80.2) | 29 (72.5) | 0.168 | 11 (64.7) | 87 (79.8) | **0.049** | 44 (81.5) | 54 (75.0) | 0.195 | 30 (76.9) | 67 (78.8) | **0.046** | 44 (84.6) | 54 (73.0) | 0.132 |
| **Peripheral PsA** | 124 (98.4) | 85 (98.8) | 39 (97.5) | 0.576 | 17 (100.0) | 107 (98.2) | 0.573 | 53 (98.1) | 71 (98.6) | 0.837 | 39(100.0) | 85(100.0) | / | 52 (100.0) | 72 (97.3) | 0.232 |
| **Axial PsA** | 54 (42.9) | 41 (47.7) | 13 (32.5) | 0.109 | 2 (11.8) | 52 (47.7) | **<0.01** | 54 (100.0) | 0 (0.0) | <0.01 | 17 (43.6) | 36 (42.4) | 0.897 | 21 (40.4) | 33 (44.6) | 0.638 |
| **Enthesitis** | 69 (54.8) | 52 (60.5) | 17 (42.5) | 0.059 | 12 (70.6) | 57 (52.3) | 0.159 | 28 (51.9) | 41 (56.9) | 0.570 | 25 (64.1) | 43 (50.6) | 0.160 | 26 (50.0) | 43 (58.1) | 0.368 |
| **Dactylitis** | 30 (23.8) | 19 (22.1) | 11 (27.5) | 0.507 | 3 (17.6) | 27 (24.8) | 0.521 | 12 (22.2) | 18 (25.0) | 0.717 | 5 (12.8) | 23 (27.1) | 0.078 | 13 (25.0) | 17 (23.0) | 0.793 |
| **Skin** | 78 (61.9) | 52 (60.5) | 26 (65.0) | 0.626 | 10 (58.8) | 63 (62.4) | 0.779 | 35 (64.8) | 43 (59.7) | 0.560 | 23 (59.0) | 54 (63.5) | 0.627 | 52 (100.0) | 27 (36.5) | **<0.01** |
| **Nails** | 44 (34.9) | 27 (31.4) | 17 (42.5) | 0.224 | 9 (52.9) | 35 (32.1) | 0.094 | 17 (31.5) | 27 (37.5) | 0.483 | 13 (33.3) | 30 (35.3) | 0.831 | 28 (53.8) | 16 (21.6) | **<0.01** |
| **Uveitis (ever).** | 6 (4.8) | 4 (4.7) | 2 (5.0) | 0.932 | 0 (0.0) | 6 (5.5) | 0.322 | 4 (7.4) | 2 (2.8) | 0.227 | 2 (5.1) | 4 (4.7) | 0.919 | 3 (5.8) | 3 (4.1) | 0.656 |
| **IBD (ever)** | 6 (4.8) | 4 (4.7) | 2 (5.0) | 0.932 | 1 (5.9) | 5 (4.6) | 0.816 | 4 (7.4) | 2 (2.8) | 0.227 | 2 (5.1) | 3 (3.5) | 0.674 | 3 (5.8) | 3 (4.1) | 0.656 |
| **DAPSA,** mean ± SD | 27.7±10.0 | 28.4±10.5 | 26.1±9.0 | 0.123 | 25.7±10.1 | 28.0±10.0 | 0.189 | 28.9±12.0 | 26.7±8.2 | 0.109 | 18.9±4.2 | 31.9±9.2 | **<0.01** | 28.5±10.4 | 27.0±9.8 | 0.203 |
| **ASDAS-CRP§,** mean ± SD | 2.65±0.69 | 2.71±0.65 | 2.46±0.8 | 0.132 | 2.7±0.28 | 2.65±0.7 | 0.457 | 2.65±0.69 | / | / | 2.59±0.64 | 2.75±0.7 | 0.140 | 2.71±0.76 | 2.61±0.64 | 0.302 |
| **Disease Duration,**  months median (IQR) | 92  (45-177) | 83 (46-177) | 103 (45-165) | 0.233 | 21 (13-55) | 103 (59-181) | **<0.01** | 108  (79-189) | 69 (31-157) | **0.038** | 106  (53-183) | 83  (45-173) | 0.085 | 103 (47-206) | 83 (45-151) | 0.111 |

Clinical features of patients’ cohort in UPREAL-PsA (Upadacitinib therapy in the real-life in patients with psoriatic arthritis)**. a-d** Cardiovascular, Metabolic, Neoplastic and Other Comorbidities are fully listed in Additional Table 1b. § Only in patients with axial PsA (n = ). PsA: Psoriatic Arthritis; PsO: Psoriasis; BMI: Body Mass Index; Ax: Axial PsA; Per: Peripheral PsA; Oligo: Oligoarticular involvement; Poly: Polyarticular Involvement; IBD: Inflammatory Bowel Disease; DAPSA: Disease Activity in Psoriatic Arthritis; ASDAS-CRP: Ankylosing Spondylitis Disease Activity Score with the C-reactive protein; SD: Standard Deviation; IQR: Interquartile Range. Statistical analysis conducted by “Stata” software- Wilcoxon test, and the p express the comparisons between the two-previous columns. P significant (in bold) if <0.05.

**Additional Table 1b.** **Characteristics of the UPREAL-PsA study patients subgroups at baseline.**

| **PsA Patients**  **Total number (%)** | | **High BMI**  **62 (49.2)** | **Norm BMI**  **64 (50.8)** | **p** | **CVD**  **40 (31.8)** | **No CVD**  **86 (68.3)** | **p** | **MTB**  **32 (25.4)** | **No MTB**  **94 (74.6)** | **p** | **High CRP**  **57 (45.2)** | **Norm CRP**  **69 (54.8)** | **p** |
| --- | --- | --- | --- | --- | --- | --- | --- | --- | --- | --- | --- | --- | --- |
| **Gender, female** | 86 (68.3) | 40 (64.5) | 46 (71.9) | 0.375 | 27 (67.5) | 59 (68.6) | 0.901 | 23 (71.9) | 63 (67.0) | 0.610 | 38 (66.7) | 48 (69.6) | 0.728 |
| **Gender, male** | 40 (31.7) | 22 (35.5) | 18 (28.1) | 0.375 | 13 (32.5) | 27 (31.4) | 0.901 | 9 (28.1) | 31 (33.0) | 0.610 | 19 (33.3) | 21 (30.4) | 0.728 |
| **Age, years ± SD** | 56.5±11.4 | 57.5±10.8 | 55.4±12.1 | 0.113 | 62.2±9.4 | 53.8±11.4 | **<0.01** | 61.2±10.7 | 54.9±11.3 | **<0.01** | 56.8±11.8 | 56.1±11.2 | 0.308 |
| **BMI, value ± SD** | 26.7±5.1 | 30.0±4.3 | 22.4±1.8 | **<0.01** | 27.2±4.9 | 26.5±5.1 | 0.232 | 29.4±5.5 | 25.8±4.6 | **<0.01** | 27.4±5.5 | 26.0±4.6 | 0.085 |
| **Cardiovascular Diseases^a^** | 40 (31.8) | 22 (35.5) | 18 (28.1) | 0.189 | 40 (100) | 0 (0) | <0.01 | 18 (56.3) | 22 (23.4) | **<0.01** | 22 (38.6) | 18 (26.1) | 0.067 |
| **Hypertension** | 27 (21.4) | 15 (24.1) | 12 (18.8) | 0.457 | 27 (67.5) | 0 (0) | <0.01 | 10 (31.3) | 17 (18.1) | 0.117 | 14 (24.6) | 13 (18.8) | 0.436 |
| **Metabolic Diseases^b^** | 32 (25.4) | 21 (33.9) | 11 (17.2) | **0.015** | 18 (45.0) | 14 (16.3) | **<0.01** | 32 (100) | 0 (0) | <0.01 | 18 (31.6) | 14 (20.3) | 0.074 |
| **Obesity** | 23 (18.3) | 23 (37.1) | 0 (0.0) | **<0.01** | 10 (25.0) | 13 (15.1) | 0.181 | 23 (71.9) | 0 (0) | <0.01 | 13 (22.8) | 10 (14.5) | 0.229 |
| **Diabetes type II** | 12 (9.5) | 9 (14.5) | 3 (4.7) | 0.060 | 8 (20.0) | 4 (4.7) | **<0.01** | 12 (37.5) | 0 (0) | <0.01 | 7 (12.3) | 5 (7.3) | 0.338 |
| **Depression/ Anxiety Disorder** | 18 (14.3) | 11 (17.7) | 7 (10.9) | 0.255 | 9 (22.5) | 8 (9.3) | **0.044** | 9 (28.1) | 9 (9.6) | **<0.01** | 9 (15.8) | 9 (13.0) | 0.344 |
| **Neoplastic Diseases^c^** | 7 (5.6) | 4 (6.5) | 3 (4.7) | 0.334 | 1 (2.5) | 6 (7.0) | 0.155 | 3 (9.4) | 4 (4.3) | 0.139 | 4 (7.0) | 3 (4.4) | 0.259 |
| **Other Comorbidities^d^** | 98 (77.8) | 50 (80.7) | 48 (75.0) | 0.225 | 32 (80.0) | 66 (76.7) | 0.323 | 26 (81.3) | 72 (76.6) | **0.019** | 47 (82.5) | 51 (73.9) | 0.227 |
| **Peripheral PsA** | 124 (98.4) | 61 (98.4) | 63 (98.4) | 0.982 | 38 (95.0) | 86 (100) | **0.037** | 30 (93.8) | 94 (100) | **0.015** | 56 (98.2) | 68 (98.6) | 0.892 |
| **Axial PsA** | 54 (42.9) | 31 (50.0) | 23 (35.9) | 0.111 | 23 (57.5) | 31 (36.0) | **0.024** | 16 (50.0) | 38 (40.4) | 0.344 | 29 (50.9) | 25 (36.2) | 0.098 |
| **Enthesitis** | 69 (54.8) | 32 (51.6) | 37 (57.8) | 0.485 | 23 (57.5) | 46 (53.5) | 0.674 | 17 (53.1) | 52 (55.3) | 0.829 | 29 (50.9) | 40 (58.0) | 0.426 |
| **Dactylitis** | 30 (23.8) | 13 (21.0) | 17 (26.6) | 0.461 | 8 (20.0) | 22 (25.6) | 0.494 | 11 (34.4) | 19 (20.2) | 0.104 | 21 (36.8) | 9 (13.0) | **<0.01** |
| **Skin** | 78 (61.9) | 40 (64.5) | 39 (60.9) | 0.552 | 23 (57.5) | 56 (65.1) | 0.411 | 19 (59.4) | 60 (63.8) | 0.653 | 40 (70.2) | 39 (56.5) | 0.082 |
| **Nails** | 44 (34.9) | 21 (33.9) | 23 (35.9) | 0.808 | 13 (32.5) | 31 (36.0) | 0.697 | 16 (50.0) | 28 (29.8) | **0.038** | 23 (40.4) | 21 (30.4) | 0.245 |
| **Uveitis (ever).** | 6 (4.8) | 4 (6.5) | 2 (3.1) | 0.381 | 2 (5.0) | 4 (4.7) | 0.932 | 3 (9.4) | 3 (3.2) | 0.156 | 3 (5.3) | 3 (4.3) | 0.810 |
| **IBD (ever)** | 6 (4.8) | 4 (6.5) | 2 (3.1) | 0.381 | 1 (2.5) | 5 (5.8) | 0.416 | 1 (3.1) | 5 (5.3) | 0.615 | 4 (7.0) | 2 (2.9) | 0.280 |
| **DAPSA,** mean ± SD | 27.7±10.0 | 30.5±10.3 | 24.9±9.0 | **<0.01** | 27.7±11.0 | 27.6±9.6 | 0.478 | 33.7±9.6 | 25.6±9.4 | **<0.01** | 29.8±9.2 | 25.9±10.4 | **0.015** |
| **ASDAS-CRP§,** mean ± SD | 2.65±0.69 | 2.74±0.73 | 2.53±0.62 | 0.132 | 2.53±0.71 | 2.74±0.67 | 0.141 | 2.74±0.7 | 2.61±0.68 | 0.255 | 2.80±0.63 | 2.52±0.72 | 0.071 |
| **Disease Duration,**  months median (IQR) | 92 (45-177) | 103 (54-177) | 79 (33-175) | 0.461 | 103 (60-170) | 83 (40-177) | 0.293 | 92 (59-153) | 91 (45-187) | 0.176 | 96 (58-177) | 83 (37-163) | 0.381 |

Clinical features of patients’ cohort in UPREAL-PsA (Upadacitinib therapy in the real-life in patients with psoriatic arthritis)**.** **a.** Ischemic Cardiomyopathy, Heart Failure, Cardiac Arrhythmias (any), valvular disease (any), Stroke, Venous Thromboembolism, Pericardial Disease, other Cardiomyopathies. **b.** Diabetes type I, Dyslipidaemia, Osteoporosis. **c.** Solid Cancer. Haematological Cancer, Non-Melanoma Skin Cancer. **d.** See Additional Table 2. § Only in patients with axial PsA (n = ). High-BMI and normal BMI: patients with body mass index >30 o <30, respectively; CVD and no CVD: patients with and without cardiovascular diseases, respectively; MTB and no MTB: patients with and without metabolic diseases, respectively; Norm- or High-CRP: C- Reactive Protein normal or upper the normal limit (0.05 mg/dl); IBD: Inflammatory Bowel Disease; DAPSA: Disease Activity in Psoriatic Arthritis; ASDAS-CRP: Ankylosing Spondylitis Disease Activity Score with the C-reactive protein; SD: Standard Deviation; IQR: Interquartile Range. Statistical analysis conducted by “Stata” software- Wilcoxon test, and the p express the comparisons between the two-previous columns. P significant (in bold) if <0.05

**Additional Table 2.** **List of the comorbidities considered as Other in patients of the UPREAL-PsA study.**

| **System / Organ Class** | **Diagnosis** | **Nr.** |
| --- | --- | --- |
| **Hematological disorders** | Anemia (syderopenic) | 1 |
|  | Monoclonal gammopathy of undetermined significance (M-GUS) | 1 |
| **Vascular disorders** | Aortic Aneurysm | 1 |
| **Gastrointestinal disorders** | Gastroesophageal reflux disease (GERD) | 5 |
|  | Gastritis (chronic) | 1 |
|  | Hepatic steatosis | 3 |
|  | Acute pancreatitis (previous) | 1 |
|  | Acute diverticolitis (previous, surgically treated) | 2 |
|  | Diverticular disease (chronic) | 2 |
|  | Gallbladder polyps (surgically treated) | 1 |
|  | Hiatal hernia | 1 |
|  | Intestinal inguinal hernia | 1 |
|  | Bowel adenoma (surgically treated) | 1 |
| **Nervous system disorders** | Multiple Sclerosis | 1 |
|  | Parkinson’s Disease | 1 |
|  | Myastenia Gravis | 1 |
|  | Sleep Disorders (Insomnia) | 1 |
|  | Neuroma (brachial plexus, surgically treated) | 1 |
|  | Schwannoma (surgically treated) | 1 |
| **Respiratory, thoracic and mediastinal disorders** | Asthma | 2 |
|  | Chronic obstructive pulmonary disease (COPD) | 4 |
|  | Pulmonary hypertension | 1 |
|  | Obstructive sleep apnea syndrome (OSAS) | 2 |
|  | Vidal Wright Syndrome | 1 |
| **Endocrine disorders** | Hyperthyroidism | 1 |
|  | Hypothyroidism | 6 |
|  | Thyroiditis (Hashimoto) | 10 |
|  | Adrenal insufficiency | 1 |
|  | Thyroid adenoma (surgically treated) | 1 |
|  | Pituitary adenoma | 1 |
| **Skin and subcutaneous tissue disorders** | Chronic urticaria | 1 |
|  | Actinic keratoses | 1 |
| **Musculoskeletal and connective tissue disorders** | Gout | 3 |
|  | Calcium pyrophosphate crystal deposition disease (CPPD) | 1 |
|  | Fybromialgia | 15 |
|  | Osteoarthritis | 2 |
|  | Spondylarthrosis | 2 |
|  | Intervertebral disc herniation with radiculopathy | 1 |
|  | Lumbar spinal stenosis | 5 |
| **Urological / Genital system disorders** | Cistitis (recurrent) | 1 |
|  | Uterine fibromatosis (surgically treated) | 2 |
|  | Uterine prolapse (surgically treated) | 1 |
| **Immune system disorders** | Schonlein-Henoch purpura (previous) | 1 |
|  | Sjogren Syndrome |  |
| **Infections and infestations** | Herpes zoster infection (previous) | 3 |
|  | Tubercolosis (previous or latent) | 3 |
|  | Hepatitis B Virus (previous infection) | 1 |
|  | Hepatitis C Virus (previous infection) | 2 |
| **Ophthalmological disorders** | Vitreous detachment | 1 |
| **Congenital / Genetic disorders** | Charcot-Marie-Tooth Syndrome | 1 |

Full list of al the comorbidities considered as “Other” (see Tables 1 – Additional Tables 1a-b) in the patients of the UPREAL-PsA (Upadacitinib therapy in the real-life in patients with psoriatic arthritis) study. Nr: number of the UPREAL-PsA patients affected.

**Additional Table 3. List of the previous bDMARDs used in bio-failure patients.**

| **bDMARD** | **n(%)** |
| --- | --- |
| Adalimumab | 76 (69.7) |
| Infliximab | 17 (15.6) |
| Certolizumab | 22 (20.2) |
| Golimumab | 36 (33.0) |
| Etanercept | 58 (53.2) |
| Ustekinumab | 21 (19.3) |
| Guselkumab | 6 (5.5) |
| Risankizumab | 2 (1.8) |
| Secukinumab | 58 (53.2) |
| Ixekizumab | 16 (14.7) |
| Tofacitinib | 5 (4.6) |
| Baricitinib | 4 (3.7) |

List of the previous biological-Disease Modifying Anti-Rheumatic Drug (b-DMARD) in the cohort of bio-experienced patients. In the right column is shown the total number of the patients (n) and the percentage of the patients (%) treated with each drug in comparison to the total number of bio-experienced patients.

**Additional Table 4.** **DAPSA responses in patients’ subgroups of the UPREAL-PsA study.**

| **Subgroups** | **Baseline** | **w12** | **p^a^** | **w24** | **p^b^** |
| --- | --- | --- | --- | --- | --- |
| Females | 28.4 ± 10.5 | 15.3 ± 10.4 | **<0.0001** | 13.2 ± 11.3 | **0.0277** |
| Males | 26,1 ± 9.00 | 11.9 ± 7.00 | **<0.0001** | 9.2 ± 9.50 | **0.0493** |
| Bio-Naive | 25.7 ± 10.1 | 9.4 ± 5.50 | **<0.0001** | 11.2 ± 14.8 | 0.5694 |
| Bio-Failure | 28.0 ± 10.0 | 14.9 ± 9.80 | **<0.0001** | 11.8 ± 9.90 | **0.0004** |
| Axial PsA | 28.9 ± 12.0 | 16.5 ± 11.8 | **<0.0001** | 13.2 ± 10.6 | **0.0008** |
| Peripheral PsA | 26.7 ± 8.20 | 12.2 ± 6.90 | **<0.0001** | 10.8 ± 10.8 | 0.0901 |
| Oligoarticular | 18.9 ± 4.20 | 10.3 ± 5.10 | **<0.0001** | 9.2 ± 6.00 | **0.0404** |
| Polyarticular | 32.0 ± 9.20 | 15.9 ± 10.3 | **<0.0001** | 13.0 ± 12.1 | **0.0213** |
| Active Pso | 28.5 ± 10.4 | 14.9 ± 9.80 | **<0.0001** | 11.2 ± 10.1 | **0.0081** |
| Inactive Pso | 27.0 ± 9.80 | 13.3 ± 9.30 | **<0.0001** | 12.0 ± 11.2 | 0.0763 |
| High CRP | 29.8 ± 9.20 | 14.5 ± 11.1 | **<0.0001** | 10.3 ± 9.70 | **<0.0001** |
| Normal CRP | 25.9 ± 10.4 | 13.6 ± 7.80 | **<0.0001** | 13.0 ± 11.6 | 0.3534 |
| High BMI | 30.5 v 10.3 | 16.2 ± 10.5 | **<0.0001** | 13.7 ± 13.1 | **0.0339** |
| Normal BMI | 24.9 ± 9.00 | 11.6 ± 7.60 | **<0.0001** | 9.1 ± 6.00 | **0.0239** |
| Cardiovascular disease Present | 27.7 ± 11.0 | 15.1 ± 7.90 | **<0.0001** | 15.0 ± 11.2 | 0.1273 |
| Cardiovascular disease Absent | 27.6 ± 9.60 | 13.6 ± 10.1 | **<0.0001** | 10.2 ± 10.3 | **0.0148** |
| Metabolic  disease Present | 33.7 ± 9.60 | 16.8 ± 9.70 | **<0.0001** | 15.6 ± 13.6 | 0.1746 |
| Metabolic  disease Absent | 25.6 ± 9.40 | 13.0 ± 9.30 | **<0.0001** | 9.9 ± 8.50 | **0.0077** |

Comparisons of the DAPSA (Disease Activity in Psoriatic Arthritis) in the UPREAL-PsA (Upadacitinib therapy in the real-life in patients with psoriatic arthritis) study at week 12(w12) and 24(w24) follow up. Bio-naïve: patients treated >3 months with conventional synthetic Disease-Modifying Anti-Rheumatic Drugs (csDMARDs); Bio-Failure: patients refractory to at least one biologic DMARDs; PsO: Psoriasis; Norm-CRP vs High-CRP: C-Reactive Protein normal or upper the normal limit (0.05 mg/dl); High- vs normal-BMI: body mass index>30 o <30, respectively. Statistical analysis conducted by “Stata” software-Wilcoxon test. P significant (in bold) if <0.05; p^a^ between baseline and w12 and p^b^ between w12 vs w24.

**Additional Table 5.** **ASDAS-CRP responses in patients’ subgroups of the UPREAL-PsA study.**

| **Subgroups** | **Baseline** | **12w** | **p^a^** | **24w** | **p^b^** |
| --- | --- | --- | --- | --- | --- |
| Females | 2.71 ± 0.65 | 1.84 ± 0.79 | **<0.0001** | 1.51 ± 0.81 | **0.0031** |
| Males | 2.46 ± 0.80 | 1.36 ± 0.72 | **0.0020** | 1.18 ± 0.68 | 0.1879 |
| Bio-Naive | 2.70 ± 0.28 | 1.90 ± 0.42 | 0.1778 | 1.60 ± 1.13 | 0.3280 |
| Bio-Failure | 2.65 ± 0.70 | 1.71 ± 0.81 | **<0.0001** | 1.42 ± 0.78 | **0.0026** |
| Oligoarticular | 2.59 ± 0.64 | 1.76 ± 0.79 | **<0.0001** | 1.54 ± 0.76 | **0.0135** |
| Polyarticular | 2.75 ± 0.70 | 1.81 ± 0.78 | **<0.0001** | 1.37 ±0. 74 | **0.0049** |
| Active Pso | 2.71 ± 0.76 | 1.76 ± 0.74 | **0.0001** | 1.50 ± 0.59 | **0.0287** |
| Inactive Pso | 2.61 ± 0.64 | 1.69 ± 0.84 | **<0.0001** | 1.41 ± 0.86 | **0.0168** |
| High CRP | 2.80 ± 0.63 | 1.73 ± 0.86 | **<0.0001** | 1.42 ± 1.02 | **0.0066** |
| Normal CRP | 2.52 ± 0.72 | 1.70 ± 0.75 | **<0.0001** | 1.44 ± 0.61 | 0.0559 |
| High BMI | 2.74 ± 0.73 | 1.75 ± 0.75 | **<0.0001** | 1.52 ± 0.73 | **0.0077** |
| Normal BMI | 2.53 ± 0.62 | 1.65 ± 0.89 | **<0.0001** | 1.28 ± 0.88 | **0.0195** |
| Cardiovascular disease Present | 2.53 ± 0.71 | 1.79 ± 0.70 | **0.0004** | 1.47 ± 0.81 | **0.0327** |
| Cardiovascular disease Absent | 2.74 ± 0.67 | 1.67 ± 0.85 | **<0.0001** | 1.40 ± 0.78 | **0.0175** |
| Metabolic  Disease Present | 2.74 ± 0.70 | 1.83 ± 0.79 | **<0.0001** | 1.54 ± 0.90 | **0.0109** |
| Metabolic  disease Absent | 2.61 ± 0.68 | 1.67 ± 0.80 | **<0.0001** | 1.36 ± 0.71 | **0.0256** |

Comparisons of the ASDAS-CRP (Ankylosing Spondylitis Disease Activity Score with the C-reactive protein) in the UPREAL-PsA (Upadacitinib therapy in the real-life in patients with psoriatic arthritis) study at week 12(w12) and 24(w24) follow up. Bio-naïve: patients treated >3 months with conventional synthetic Disease-Modifying Anti-Rheumatic Drugs (csDMARDs); Bio-Failure: patients refractory to at least one biologic DMARDs; PsO: Psoriasis; Norm-CRP vs High-CRP: C-Reactive Protein normal or upper the normal limit (0.05 mg/dl); High- vs normal-BMI: body mass index>30 o <30, respectively. Statistical analysis conducted by “Stata” software-Wilcoxon test. P significant (in bold) if <0.05; p^a^ between baseline and w12 and p^b^ between w12 vs w24.

**Additional Table 6.** **List of all the adverse events observed in patients of the UPREAL-PsA study**.

| **System Organ Class** | **CTCAE** | **Grade** | **Nr.** |
| --- | --- | --- | --- |
| Gastrointestinal Disorders | Nausea | Grade 1 – mild | 1 |
|  | Diarrhea | Grade 1 – mild | 2 |
|  | Dyspepsia | Grade 1 – mild | 1 |
| Vascular Disorders | Hypertension | Grade 3 – severe | 1 |
|  | Hypotension | Grade 1 – mild | 1 |
| Skin and subcutaneous tissue disorders | Bullous dermatitis | Grade 2 – moderate | 1 |
| Infections and infestations | Herpes simplex reactivation | Grade 1 – mild | 2 |
|  | Urinary tract infection | Grade 2 – moderate | 2 |
|  | Sinusitis | Grade 2 – moderate | 1 |
| Investigations | Alanine aminotransferase & aspartate aminotransferase increased | Grade 1 – mild | 1 |
|  |  | Grade 2 – moderate | 1 |
|  | Neutrophil count decreased | Grade 2 – moderate | 1 |
|  | Platelet count decreased | Grade 2 – moderate | 2 |
|  | CPK increased | Grade 2 – moderate | 1 |
|  |  | Grade 3 – severe | 1 |
| SARS-CoV-2 related | SARS-CoV-2 Infection | Grade 2 – moderate | 2 |

In the table are shown all the adverse events reported during the UPREAL-PsA (Upadacitinib therapy in the real-life in patients with psoriatic arthritis) study until week 24 of follow up and classified according to Common Terminology Criteria for Adverse Events (CTCAE) version 5.0. CPK: Creatine Phospho-Kinase; SARS-CoV-2: Severe Acute Respiratory Syndrome Coronavirus 2.
