# Supplementary figures and images for: Upadacitinib effectiveness and factors associated with minimal disease activity achievement in patients with psoriatic arthritis: preliminary data of a real-life multicenter study
Source: Arthritis Res Ther. 2023 Oct 11;25:196. doi: 10.1186/s13075-023-03182-9 (PMC10565976; doi:10.1186/s13075-023-03182-9)

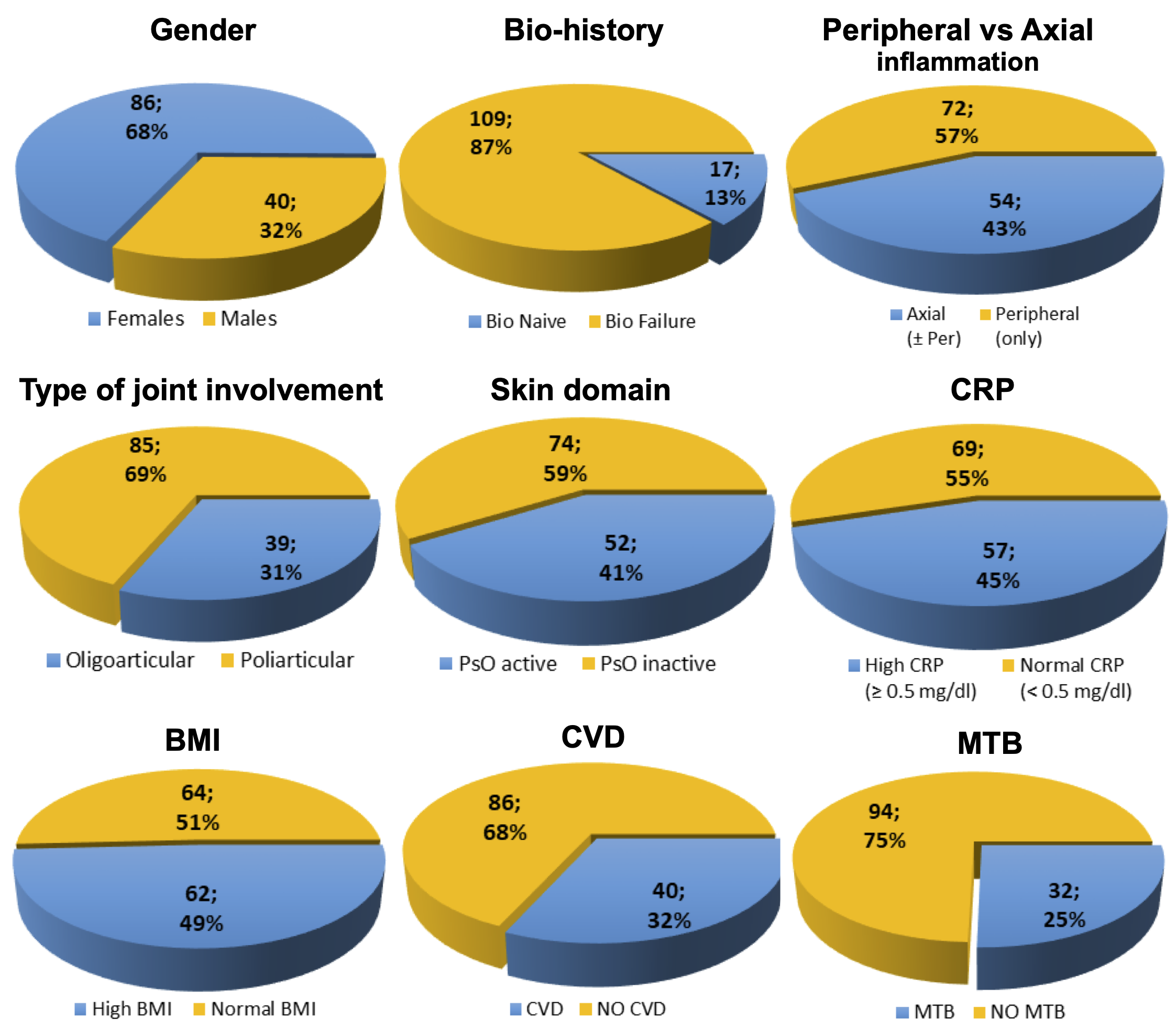

Supplement: Supplementary file 2 — Additional file 2: Additional Figure 1. Characteristics of the UPREAL-PsA patients at baseline. Graphical representation of the most representative clinical features of the patient’s cohort of the UPREAL-PsA (Upadacitinib therapy in the REAL life of patients with psoriatic arthritis) study. Bio-naïve: patients treated>3 months with conventional synthetic Disease-Modifying Anti-Rheumatic Drugs (csDMARDs); Bio-Failure: patients refractory to at least one biologic DMARDs; PsO: Psoriasis; Norm- or High-CRP: C- Reactive Protein normal or upper the normal limit (0.05 mg/dl); High-BMI and normal BMI: patients with body mass index>30 o <30, respectively; CVD: cardiovascular comorbidities and MTB: Metabolic Diseases: see the full list in Table 1. [file 13075_2023_3182_MOESM2_ESM.tiff]
